# Spatial constraints within the chlamydial host cell inclusion predict interrupted development and persistence

**DOI:** 10.1186/1471-2180-8-5

**Published:** 2008-01-09

**Authors:** Alexander Hoare, Peter Timms, Patrik M Bavoil, David P Wilson

**Affiliations:** 1 National Centre in HIV Epidemiology and Clinical Research, Faculty of Medicine, University of New South Wales, Sydney, NSW, Australia; 2 Institute of Health and Biomedical Innovation, Queensland University of Technology, Brisbane, QLD, Australia; 3 Department of Biomedical Sciences, University of Maryland Baltimore, MD, USA

## Abstract

**Background:**

The chlamydial developmental cycle involves the alternation between the metabolically inert elementary body (EB) and the replicating reticulate body (RB). The triggers that mediate the interchange between these particle types are unknown and yet this is crucial for understanding basic *Chlamydia *biology.

**Presentation of the hypothesis:**

We have proposed a hypothesis to explain key chlamydial developmental events whereby RBs are replicating strictly whilst in contact with the host cell membrane-derived inclusion via type three secretion (T3S) injectisomes. As the inclusion expands, the contact between each RB and the inclusion membrane decreases, eventually reaching a threshold, beyond which T3S is inactivated upon detachment and this is the signal for RB-to-EB differentiation.

**Testing the hypothesis:**

We explore this hypothesis through the development of a detailed mathematical model. The model uses knowledge and data of the biological system wherever available and simulates the chlamydial developmental cycle under the assumptions of the hypothesis in order to predict various outcomes and implications under a number of scenarios.

**Implications of the hypothesis:**

We show that the concept of *in vitro *persistent infection is not only consistent with the hypothesis but in fact an implication of it. We show that increasing the RB radius, and/or the maximum length of T3S needles mediating contact between RBs and the inclusion membrane, and/or the number of inclusions per infected cell, will contribute to the development of persistent infection. The RB radius is the most important determinant of whether persistent infection would ensue, and subsequently, the magnitude of the EB yield. We determine relationships between the length of the T3S needle and the RB radius within an inclusion, and between the RB radius and the number of inclusions per host cell to predict whether persistent infection or normal development would occur within a host cell. These results are all testable experimentally and could lead to significantly greater understanding of one of the most crucial steps in chlamydial development.

## Background

Chlamydiae are bacterial pathogens of very significant public health concern due to extensive morbidity, especially associated with female reproductive health. Infectious chlamydial particles (known as elementary bodies (EBs)) attach to, and internalize within, host eukaryotic cells. An infecting EB will be internalized in a plasma membrane-derived vacuole of the host cell (known as an inclusion). Soon after internalization, the EB undergoes morphological changes, differentiating into its replicative form, the reticulate body (RB), and RBs double their DNA content approximately every 2–3 hours upon binary fission [1-3]. After 6 to 10 rounds of replication, infectious EBs appear as the RBs convert to EBs while some RBs continue to replicate. Late in development, the majority of RBs are differentiating into EBs until the host cell lyses, releasing the infectious EB particles for subsequent rounds of infection of new cells. Whilst this developmental cycle is reasonably well characterized morphologically, the molecular and cellular signals that trigger the differentiation of EB-to-RB and RB-to-EB are unknown. Under certain stressful conditions, dividing RBs transition from their normal state to very large, morphologically aberrant maxi-RBs (mRBs) which are non-infectious, have limited capacity to divide, and do not convert to EBs [4,5] (thus, the regular lytic cycle is halted indefinitely). Such a persistent state can be induced during *in vitro *infection with agents such as penicillin, IFN-γ or by nutrient deprivation [6]. Although increasing evidence through continuous growth models suggests that this phenomenon occurs during *in vivo *infection [7,8], we refer here to persistence as it occurs *in vitro*. In this paper we do not distinguish between the various conditions and mechanisms that contribute to inhibition of RB division, abnormalities in RB size, or different numbers of host cell inclusions. Here, we investigate a hypothesis and its theoretical implications given such geometric properties and morphologic abnormalities. Thus, our results are not contingent on the actual conditions or mediators leading to the abnormalities but suggest how the abnormalities influence development or persistence.

## Presentation of the hypothesis

Our hypothesis is an attempt to explain the mid-to-late cycle transitions of *Chlamydia *[9]. The hypothesis is that:

(i) RBs grow strictly in contact with the plasma membrane-derived chlamydial inclusion membrane (CIM);

(ii) Contact is mediated by type three secretion (T3S) injectisomes proposed to correspond to surface projections previously described by Matsumoto [10],

(iii) As the CIM grows, T3S activity decreases per RB until the RB detaches from the CIM, and that this detachment from the CIM constitutes the signal for late RB-to-EB differentiation.

We initially introduced this hypothesis with a simple mathematical model. Here, we predict greater implications of our hypothesis by considering various geometric aspects (how the size of RBs, EBs and the inclusion can affect persistence) and stochastic aspects (how random biological processes of individual RB detachment and RB-to-EB differentiation influence developmental dynamics). We show that the seemingly distinct phenomenon of chlamydial *in vitro *persistence is not only consistent with our contact dependent hypothesis but an implication of it. We make experimentally testable predictions of specific criteria distinguishing persistent infections from normal development.

## Testing the hypothesis

We make predictions of the conditions under which normal or interrupted (i.e. persistent infection) development would occur through the development and analysis of a novel mathematical model; it is the most advanced within-host model of *Chlamydia *and as far as we are aware it is the only model to describe geometric features of intracellular pathogen growth and to explicitly model particles in the process of replication and division. Our model tracks individual bodies and stochastic variations that can occur within a cell, which is significantly more biologically realistic than previous models. We investigate stochastic and deterministic aspects of the model (see Additional file 1), simulating infection dynamics with second order Monte Carlo simulations based on Latin Hypercube Sampling [11], and we perform multivariate sensitivity analyses on the model outcomes to draw relationships between parameters and infection outcomes; Table 1 lists definitions and values of all parameters of the model. The stochastic aspects of the model allow us to investigate the random processes of individual chlamydial bodies (e.g., detachment of RBs, and the differentiation of RBs to EBs). This provides a more natural model framework and more accurately tracks low numbers of RBs and EBs in the case of persistent infections. The deterministic model works well for large numbers of EBs and RBs, it does not include any random processes (thus it is much simpler than a stochastic model), and it can be used to perform mathematical analyses. Both types of models incorporate the same geometric aspects of RBs and inclusions. We vary all infection parameters over a wide parameter space to account for the biological heterogeneity and to assess the variability in the outcomes as determined under a range of different conditions.

**Table 1:** Parameter definitions and ranges used in our biomathematical simulations

**Parameter**	**Definition**	**Range**	**References**
*t*_*d*_	Average doubling time of RBs during phase of exponential growth (replication and division of bodies)	1.5–2.6 hrs	[1-3]
*α*_0_	Proportion of total doubling time in which RB genomes are dividing into separate bodies during uninhibited exponential growth	0.1–0.5	
1/*k*	Average time for RB detachment from the inclusion membrane and migration to the lumen to occur, when the number of injectisomes is half the threshold level	0.01–1.0 hrs	†
1/*μ*	Average time for RB-EB differentiation	1–4 hrs	[2, 3, 38]
*L*	Average center-to-center spacing between T3S needles on the RB surface	40–50 nm	[10, 32]
*l*_*p*_	Average length of each needle	5–10 nm	§ [33]
*r*	Radius of each RB (without stress)	0.5–0.6 μm	[38]
*r*_*E*_	Radius of each EB	0.1–0.2 μm	[38]
Cell_*vol*_	Volume of host cell	1700–2500 μm^3^	Experimental Variable
*ε*	Proportion of cell space occupied by cell nucleus, mitochondria, etc.	0.2–0.4	Experimental Variable
*V*_*I*_	Volume within the lumen occupied per detached RB (considering effects of steric hindrance)	*V*_*I *_= (2*r*)^3^	
*V*_*E*_	Volume within the lumen occupied per EB (considering effects of steric hindrance)	*V*_*E *_= (2*r*_*E*_)^3^	
*P*_lim_	Threshold number of injectisomes per RB for the detachment of RBs from the inclusion membrane	21–25	[9]

†: Experimental variable. It was calibrated so that it matched observational experimental time courses.

§: The full length of the needle has been measured to be 10–25 nm, but we assume that the length of the needle between the RB and the CIM required to establish effective contact is 5–10 nm.

**Figure 1:**
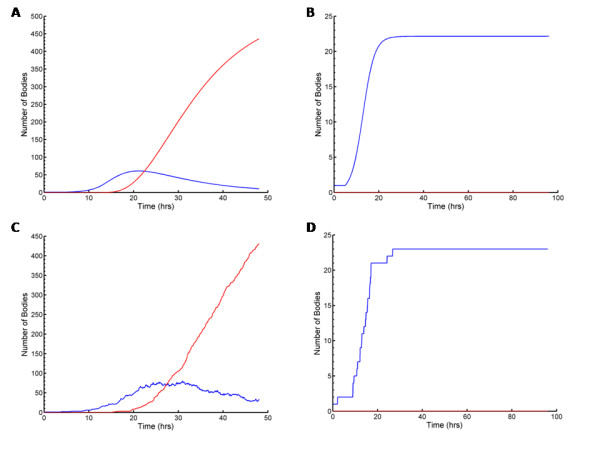
Typical time course plots resulting from simulation with our mathematical models; the red curves represent the number of EBs and the blue curves represent the number of RBs. We run the deterministic model to produce **(a) **normal development (with the doubling time *t*_d _= 1.8 hrs, rate of RB detachment *k *= 1.3 hrs^-1^, EB radius *r*_e _= 0.1 *μ*m, rate of RB-to-EB differentiation *μ *= 0.25 hrs^-1^, average spacing between T3S needles on the RB surface *L *= 0.04 *μ*m, length of T3S needles *l*_*p *_= 0.0078 *μ*m, volume of host cell Cell_Vol _= 2400 *μ*m^3^, proportion of cell not available for inclusion growth *ε *= 0.3, threshold number of T3S needles per RB *P*_lim _= 23, number of inclusions *N *= 1, and RB radius *r *= 0.5 *μ*m) and **(b) **persistent infection (by increasing only the size of the RB radius, *r *= 2.0 *μ*m). Figures **(c) **and **(d) **show simulations of the stochastic model, using the same parameter values as used in (a) and (b) respectively.

**Figure 2:**
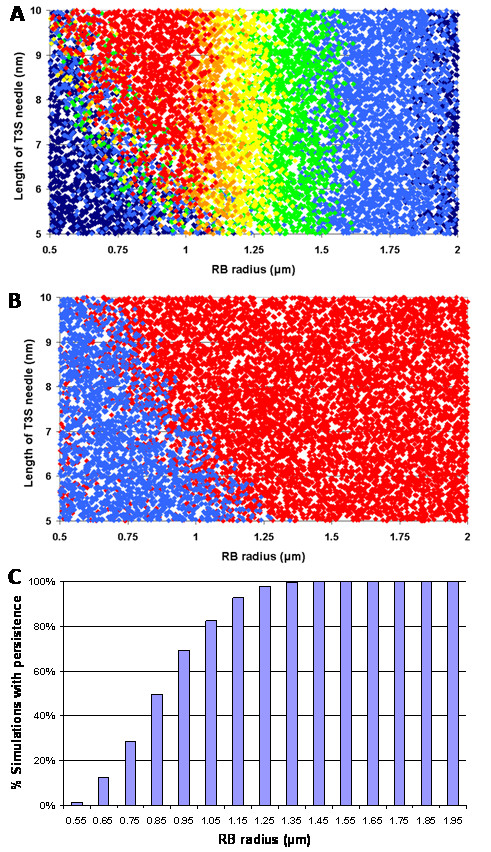
Scatterplot from 10,000 stochastic model time course simulations of the length of T3S needle versus the RB radius, color-coded **(a) **for the maximum number of RBs, *RB*_max_: dark blue *RB*_max _< 20, light blue 20 ≤ *RB*_max _< 40, green 40 ≤ *RB*_max _< 60, yellow 60 ≤ *RB*_max _< 80, orange 80 ≤ *RB*_max _< 100, and red *RB*_max _> 100; **(b) **by whether normal (blue) or interrupted (red) development, leading to persistent infection occurred. **(c) **Histogram summarizing 10,000 simulations of persistence or normal development, grouped by the size of RB radius; groupings are in intervals of 0.1 *μ*m and the mid-point of the interval is shown.

Our mathematical model accurately reflects normal development time courses which have been previously observed experimentally (Figure 1). The stochastic version of our model is essentially equivalent to the deterministic version (compare Figs. 1a,c and 1b,d); the only difference between the models is that the stochastic version includes the inherent randomness of the biological events that occur in development and thus for any given set of parameters different outcomes will be produced, whereas the deterministic version will always produce the same (smooth) outcome. Our model clearly predicts that by increasing only the size of the RB radius (from 0.5 *μ*m to 2.0 *μ*m) normal development is interrupted, leading to a persistent mode of growth (Figure 1). Indeed, multivariate sensitivity analyses revealed that of all the factors that contribute to intracellular chlamydial development, the RB radius was the most important parameter influencing the outcome of infection, followed by the maximum length of the T3S needle mediating contact between the RB and CIM (i.e. the T3S substructure delimiting the distance between the chlamydial outer membrane anchor and the T3S-secreted translocator proteins in the inclusion membrane), and then the number of inclusions (not shown). We carried out 10,000 stochastic simulations of chlamydial infection and varied all parameters with each simulation. We determined that for large RB radius (> ~1.2 *μ*m) the length of the T3S needle did not influence development, but for smaller RB radii, development was dependent upon both RB radius and needle length (< ~1.2 *μ*m) (Fig. 2a). In this case, longer needles resulted in greater maximum numbers of RBs and consequently greater EB yield (Fig. 2a). This is because smaller RBs have greater contact with the CIM (e.g. in areas where the CIM-RB distance is greatest, such at the edge of the contact area), and thus the RBs continue replicating longer before the detachment threshold is reached. Similarly, larger RBs also resulted in greater numbers of chlamydial particles generated (Fig. 2a). However, there is a maximal possible number of particles generated after which the number of particles produced starts to decrease with RB radius. This switch between the increase and decrease in chlamydial particles produced occurs at the threshold between normal and interrupted development, i.e. persistence (Fig. 2b). In Figure 2b simulations were color-coded according to interrupted (red) or normal development (blue); this clearly demonstrates a relationship between needle length and RB radius that together defines the predicted threshold for the outcome of infection. The threshold is physically interpreted as the point at which, for a given sized inclusion, attachment strength has decreased where a spectrum of RBs ranging from large RBs with shorter needles to small RBs with longer needles, are on the verge of detachment. Notwithstanding the impact of needle length, the RB radius was the most important determinant of the outcome of infection; thus we explored the percentage of simulations resulting in persistent infection versus the RB radius (Fig. 2c). Persistence increased continuously with RB radius and persistence occurred in all simulations where the RB radius was greater than 1.3 *μ*m.

**Figure 3:**
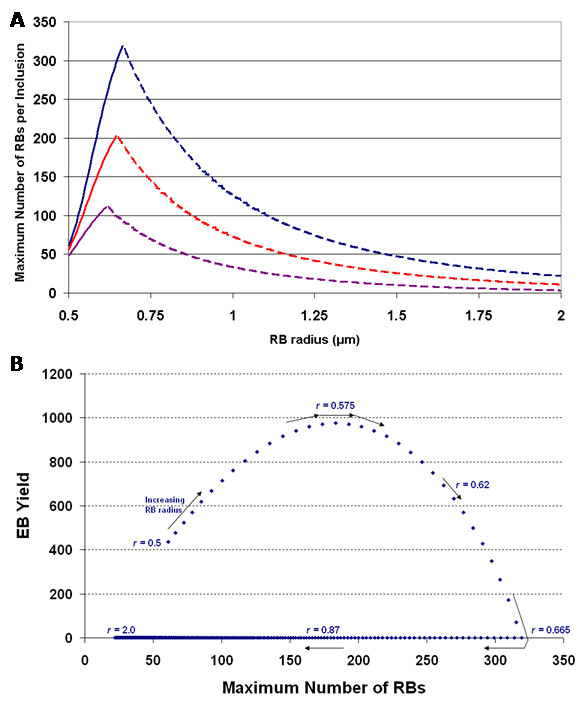
**(a) **The maximum number of RBs attained per inclusion versus RB radius size for one inclusion (blue), two inclusions (red), and five inclusions (magenta). The solid section of the curves corresponds to normal development and the dashed section corresponds to interrupted development leading to a persistent mode of growth. **(b)** The relationship between EB yield and the maximum number of RBs within a single inclusion. Each point represents a different value for the RB radius, with the radius increasing in the direction of the arrows. Parameter values used were: doubling time *t*_d _= 1.8 hrs, rate of RB detachment *k *= 1.3 hrs^-1^, EB radius *r*_e _= 0.1 *μ*m, rate of RB-to-EB differentiation *μ *= 0.25 hrs^-1^, average spacing between T3S needles on the RB surface *L *= 0.04 *μ*m, length of T3S needles *l*_*p *_= 0.0078 *μ*m, volume of host cell Cell_Vol _= 2400 *μ*m^3^, proportion of cell not available for inclusion growth *ε *= 0.3, and threshold number of T3S needles per RB *P*_lim _= 23.

Multiple inclusions will reduce the space available within the host cell for each inclusion to grow; i.e., they will in effect reduce the size of each inclusion, and this effectively shifts the threshold curve. We expect that persistence is more likely to occur with greater numbers of inclusions. This is exactly what we observe from our model simulations. We explored the effect of the size of the RB radius on the maximum number of RBs produced per inclusion and also varied the number of inclusions. In Figure 3a we present the maximum number of RBs versus RB radius for the cases of 1 inclusion (blue), 2 inclusions (red), and 5 inclusions (magenta). The solid sections of the curves refer to normal development and the dashed sections refer to interrupted development, leading to persistent infection. We note that the profile for the number of chlamydiae produced increases with RB radius until the point of interrupted development and then decreases (Fig. 3a). The increase with larger RBs is due to increased surface area available for contact with the CIM and thus the inclusion must grow larger, accommodating more RBs, before the threshold contact is reached. If normal development is interrupted, lower numbers of larger RBs will result. This is because growth in the persistent mode coincides with spatial limitations in the host cell being reached; fewer numbers of larger bodies can physically fit in the cell. Increasing the number of inclusions per cell reduces the maximal number of RBs per inclusion (as expected), but more importantly interrupted development is observed to occur for smaller RBs (Fig. 3a) leading to their switching to a persistent mode. The curves in Figure 3a were generated from the deterministic model, setting all parameters to the median values over the range. This resulted in persistence with one inclusion once the RB radius reached 0.665 *μ*m (peak number of RBs is 320). However, this critical RB size decreases to 0.645 *μ*m (203, peak number of RBs) with two inclusions and to 0.615 *μ*m with 5 inclusions (111, peak number of RBs). Interestingly, the number of EBs resulting from infection does not mirror the maximal number of RBs but there is a strong non-linear relationship (Fig. 3b). The maximal number of RBs is greatest when normal development is on the verge of interruption; therefore, this situation results in no EBs being produced. There is an optimal RB radius (0.575 *μ*m) for producing the greatest EB yield (Fig. 3b). Lower than the optimal RB radius, RBs start detaching from the CIM faster and thus do not produce as many (intermediate) bodies with the potential of differentiating to EBs. In contrast, RBs with a radius greater than the optimal remain attached to the CIM for longer and the cell will fill up with chlamydiae before all the RBs have differentiated to EBs. Thus, after the optimal RB radius, increasing the radius results in decreased numbers of EBs and increased numbers of RBs until a persistent mode of growth results (number of EBs produced is zero, Fig. 3b). After this point, increasing the RB radius also decreases the number of RBs produced (Fig. 3b).

**Figure 4:**
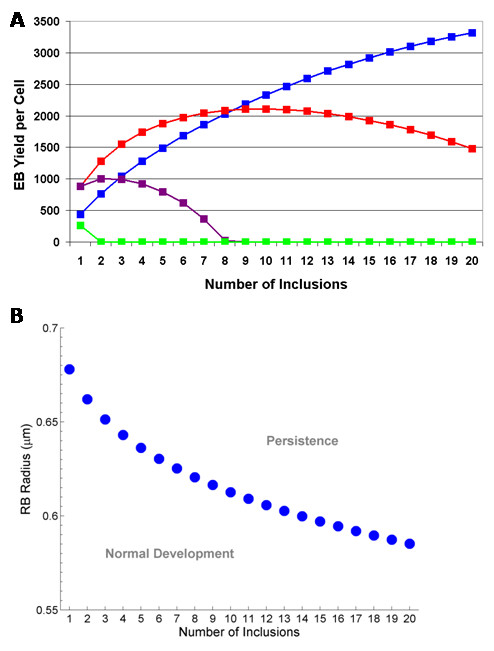
**(a) **The effect of the number of inclusions on the EB yield; average RB radii of 0.5 *μ*m (blue), 0.55 *μ*m (red), 0.6 *μ*m (magenta), and 0.65 *μ*m (green). **(b) **Threshold curve distinguishing normal development or persistent mode of growth as predicted by the size of the RB radius and the number of inclusions. Parameter values used were: doubling time *t*_d _= 1.8 hrs, rate of RB detachment *k *= 1.3 hrs^-1^, EB radius *r*_e _= 0.1 *μ*m, rate of RB-to-EB differentiation *μ *= 0.25 hrs^-1^, average spacing between T3S needles on the RB surface *L *= 0.04 *μ*m, length of T3S needles *l*_*p *_= 0.0078 *μ*m, volume of host cell Cell_Vol _= 2400 *μ*m^3^, proportion of cell not available for inclusion growth *ε *= 0.3, and threshold number of T3S needles per RB *P*_lim _= 23.

The EB yield of a *Chlamydia*-infected cell is highly dependent on the RB radius and the number of inclusions. In Figure 4a we present the EB yield versus the number of inclusions for average RB radii of 0.5 *μ*m (blue), 0.55 *μ*m (red), 0.6 *μ*m (yellow), and 0.65 *μ*m (green). For normal sized RBs (~0.5–0.6 *μ*m) there is an increase in the EB yield for an increase in inclusion numbers until some optimal level, after which point the EB yield decreases toward no EBs (that is, persistence). We note that for small RBs (0.5 *μ*m) the optimal number of inclusions is predicted to have not been reached after 20 inclusions; thus persistence is very unlikely to occur with the smaller RBs (Fig. 4a). A slightly larger RB (~0.55 *μ*m) will produce its greatest EB yield with ~9 inclusions and any more inclusions will decrease the yield towards a persistent mode of growth. The optimal number of inclusions for larger RBs will be reached with fewer inclusions. For a large RB (≥ ~0.65 *μ*m) the maximum EB yield occurs with one inclusion (Fig. 4a). If RBs have a radius of 0.65 *μ*m and there is one inclusion, normal development will ensue but if there are two inclusions, persistence will be the outcome. Therefore, to distinguish normal development from persistent growth we calculated, from our deterministic model, an analytical curve for the threshold between these cases. In Figure 4b we display this threshold curve. Persistence is more likely with greater RB radius and with more inclusions. The relationship we present in Figure 4b requires experimental validation. But it appears to be consistent with experimental observations and it is the quantitative logical conclusion of our T3S contact-dependent hypothesis for modulation of chlamydial development.

## Implications of the hypothesis

It is hypothesized that chlamydial particles have T3S injectisomes and that the *Chlamydia *T3S system functions as a molecular nanosyringe involved in injecting effector proteins from the intracellular inclusion into the host cell cytosol which, in turn, subvert various host cell pathways [12-15]. RBs are observed to replicate in close proximity to the surface of the host inclusion membrane [16,17] and as an inclusion expands (to accommodate the growth in the number of chlamydiae), the surface area of contact between the inclusion membrane and RBs on the inclusion membrane surface decreases. Consequently, the number of injectisomes per RB in contact with the inclusion membrane decreases. We have hypothesized that when the decreasing number of injectisomes on the RB surface that are in contact with the inclusion membrane falls below a threshold number, detachment of the RB from the surface occurs, providing a signal for differentiation of RB into EB.

A wide range of Gram negative bacteria have the T3S system including *Yersinia*, *Salmonella*, *Shigella*, *Escherichia*, *Pseudomonas*, *Bordetella*, *Burkholderia*, a variety of plant pathogens, and of course *Chlamydia *[18]. Possible T3S-mediated functions include the intracellular survival of *Chlamydia *in the acquisition of nutrients, inhibiting fusion with host cell lysosomes, and diverting lipids. T3S activity as regulated by contact between the RB and the inclusion membrane supports the hypothesis that T3S inactivation is a possible trigger for late differentiation and it would explain why chlamydial development, initially almost synchronous, becomes asynchronous in the mature inclusion as reticulate bodies differentiate into elementary bodies [19]. Furthermore, our T3S hypothesis for chlamydial development is not only consistent with, but predicts, persistent infection under various conditions.

There is a large portion of the *Chlamydia *literature that describes chlamydial persistence (a topic that had seemed mutually exclusive to T3S). Persistent maxi-RBs express unique RNA and protein profiles, including reduced amounts of the chlamydial MOMP, a potential protective antigen [20], and increased levels of the chlamydial heat shock proteins [4,21], which correlates with the pathological serious disease sequelae associated with chlamydial infections [22,23]. We have shown quantitatively how our hypothesis is consistent with the atypical persistent model. According to our hypothesis, chlamydiae can persist, that is, remain in RB form for long periods of time, by maintaining a number of injectisomes above a detachment threshold. There are two conditions by which this will occur: (i) if the inclusion becomes very small (e.g., small, dividing inclusions) or (ii) if the RBs become relatively very large (e.g, under stress RBs do not divide). This is precisely what happens in the persistence models. In persistent models *in vitro*, the maxi-RBs are larger and the inclusions are actually much smaller (especially in *C. pneumoniae *models). Here, a larger RB in a smaller inclusion would necessarily make more contact with the membrane, hence would maintain a sufficient number of T3S needles and never fall off, hence never differentiate. In continuous growth models, there are often many inclusions per host cell and they are of different sizes. Some inclusions are relatively small and would include only a few chlamydiae (in a persistent state). Infection by certain *Chlamydia *species, such as *C. pneumoniae *or many veterinary isolates, consistently results in multiple inclusions that do not fuse [19,24]. Further, in cases where infected host cells may still have the ability to divide such that the inclusions are distributed between the daughter cells, then small persistent inclusions would potentially have the ability to persevere for an extended period of time. We must make special note that we are not suggesting any causality in the mode for inducing persistence in terms of the external conditions that lead to spatial constraints. But we do hypothesize that if external or infection conditions, or even potentially stochasticity, contribute to larger sizes of RBs (including their inability to divide), to multiple chlamydial inclusions, or to other spatial constraints, that these geometric effects are then what inhibits detachment of RBs and triggering for differentiation into EBs. Our simulation analysis suggests that the relationships we established between the various geometrical attributes could be expected to be equivalent across various models of persistence. Because different persistent models interrupt development in various ways, the influence of the stress on all geometric factors could differ and must be considered, but our threshold relationships should be robust across models. Further, the size of RBs under *in vitro *stressed conditions, such as the addition of penicillin, may depend on the number of RBs present when the stressor is added. Our results are not influenced by the observed physical relationship between the number of RBs and their size, but our results are essentially based on the initial condition of a certain number of RBs, their size, and other geometric properties of the system in predicting whether or not RB detachment and differentiation will occur.

In this study we have analyzed chlamydial infection and predicted the conditions that will lead to persistence or normal development, according to our T3S contact-dependent hypothesis. Our investigation was carried out through the development of a novel mathematical model that extensively advanced previous similar analyses. Modeling can predict specific relationships between pathogen characteristics and infection parameters and it can determine threshold levels critical for development. We have included considerable detail of geometrical features and investigated a deterministic ordinary differential equation model and also a detailed stochastic analog of these equations. This level of detail was useful in accurately modeling persistent infection. As far as we are aware this is the first time that bacterial development and replication have been modeled in such detail. This model is useful not only to verify the plausibility of the T3S contact dependent hypothesis of development, but the quantification also produces experimentally testable predictions regarding the threshold between normal development and persistence.

The study of chlamydial biology is reputedly difficult because of the superimposed complexity of the obligate intracellular and developmental life cycle, and the genetic intractability of the organisms. In practical terms, this means that genetic characterization is limited to sequence analysis, that a molecular Koch postulate-type analysis for virulence genes is not possible, and that any attempt at molecular characterization suffers from the constant threat of contamination by host cell components. Alternative strategies such as biomathematical modeling therefore provide avenues for developing testable hypotheses that could not be obtained otherwise. Experimental verification of the predicted model results can form the basis for further modeling which may in turn generate motivation for further experimentation. Several lines of experimentation should be undertaken based on the results of the biomathematical model. For example, a prediction is that the length of the T3S needle directly impacts detachment of the RB from the inclusion membrane. Since in other pathogens the length of the T3S needle is tightly controlled and length is directly related to T3S activity [25-29], this concept begs for experimental verification in *Chlamydia*, for example using newly developed cryo-transmission electron microscopy or tomography methodologies. Indeed a tantalizing possibility is that the needle length varies during development and/or even between *Chlamydia *species. Another prediction from this study is that the number of inclusions per cell is directly related to the productive versus persistent growth outcome. Spontaneously arising mutations of the chlamydial gene *incA*, encoding an inclusion membrane protein, have been described that are altered in inclusion fusogenicity within the infected cell [24,30,31]. Although these do not represent true isogenic mutants, a comparison of the normal versus persistent growth outcome using standard methodologies in these variants compared with their "wild-type" counterparts would be very worthwhile. The fact that the *incA *gene product is itself a T3S effector protein makes the case for this phenotypic comparative analysis even more compelling.

Our modeling has shown that increasing the RB radius, or the length of needles mediating contact between RBs and the CIM, or the number of inclusions, will contribute to the establishment of the persistent mode of growth. The RB radius is the most important determinant for deciding if persistent infection will ensue and we predict that it will always occur if the average RB radius is greater than ~1.3 *μ*m. The RB radius is also the main determinant of the EB yield and there is an optimal RB radius for producing the greatest EB yield. However, the EB yield is also dependent on the number of inclusions. Together, there is a relationship between the RB radius and the number of inclusions that predicts whether persistent growth or normal development will occur within a host cell. Within an inclusion, the RB radius and the length of the T3S needles determine the level of established contact between the RB and CIM and the level of T3S activity. We calculated an analytical curve for the threshold between infection outcomes and also predicted the maximum RB numbers and EB yield based on these variables.

We were initially surprised that the length of the T3S needles was as sensitive as it was towards the outcome of infection. The physical structure, size, and density of the needles on the chlamydial surface are crucial and we strongly recommend that experiments be performed to obtain measurements of these quantities over the developmental cycle for several *Chlamydia *species. In the early 1980's Matsumoto published a series of papers providing such quantities for surface projections of *Chlamydia psittaci *strain CAL-10 [10,32,33] that have since been proposed to correspond to the T3S injectisomes and their outermost needle structures. This work was carried out long before T3S was discovered and the results were not utilized for the following two decades. However, the experimental measurements have not been repeated and although surface projections have been observed on other chlamydial species [34-37] quantitative measurements have not yet been obtained. Greater understanding of chlamydial T3S and the contact relationship between the RB and the CIM is required. Experiments should also be designed to test all the outcomes predicted in this study. *In vitro *experiments can be used to match against the threshold relationships presented in this study. These threshold curves are easily understandable and the relationships can be tested. If the hypothesis and model outcomes are verified experimentally, then the data obtained can be used to further fine-tune the model and greater complexity can also be introduced, leading to further predictions.

## List of abbreviations

EB: Elementary Body

RB: Reticulate Body

mRB: maxi-RB

T3S: Type Three Secretion

CIM: Chlamydial Inclusion Membrane

MOMP: Major Outer Membrane Protein

## Authors' contributions

AH developed geometrical features of the model, developed computer code and algorithms to implement the mathematical models, conducted mathematical analysis, produced numerical simulations, generated results, and contributed to writing the methods. PT contributed to interpreting results and editing the manuscript. PMB conceived the initial hypothesis and contributed to interpretation of results and implications and writing of the manuscript. DPW developed the concept, design and methodology for this study, conducted mathematical analysis, interpreted results, wrote the manuscript, and supervised the project.

## Supplementary Material

Additional file 1 Details of modeling methodology and algorithms. Provides detailed information to describe the mathematical modelling methodology (including deterministic equations, geometry, and algorithms for stochastic simulations). Click here for file

## References

[B1] Mathews SA, Volp KM, Timms P (1999). Development of a quantitative gene expression assay for Chlamydia trachomatis identified temporal expression of sigma factors. FEBS Lett.

[B2] Shaw EI, Dooley CA, Fischer ER, Scidmore MA, Fields KA, Hackstadt T (2000). Three temporal classes of gene expression during the Chlamydia trachomatis developmental cycle. Molecular microbiology.

[B3] Wilson DP, Mathews S, Wan C, Pettitt AN, McElwain DL (2004). Use of a quantitative gene expression assay based on micro-array techniques and a mathematical model for the investigation of chlamydial generation time. Bulletin of mathematical biology.

[B4] Beatty WL, Belanger TA, Desai AA, Morrison RP, Byrne GI (1994). Tryptophan depletion as a mechanism of gamma interferon-mediated chlamydial persistence. Infect Immun.

[B5] Harper A, Pogson CI, Jones ML, Pearce JH (2000). Chlamydial development is adversely affected by minor changes in amino acid supply, blood plasma amino acid levels, and glucose deprivation. Infection and immunity.

[B6] Hogan RJ, Mathews SA, Mukhopadhyay S, Summersgill JT, Timms P (2004). Chlamydial persistence: beyond the biphasic paradigm. Infection and immunity.

[B7] Perry LL, Feilzer K, Caldwell HD (1997). Immunity to Chlamydia trachomatis is mediated by T helper 1 cells through IFN-gamma-dependent and -independent pathways. J Immunol.

[B8] Rottenberg ME, Gigliotti-Rothfuchs A, Wigzell H (2002). The role of IFN-gamma in the outcome of chlamydial infection. Curr Opin Immunol.

[B9] Wilson DP, Timms P, McElwain DL, Bavoil PM (2006). Type III secretion, contact-dependent model for the intracellular development of chlamydia. Bulletin of mathematical biology.

[B10] Matsumoto A (1982). Electron microscopic observations of surface projections on Chlamydia psittaci reticulate bodies. Journal of bacteriology.

[B11] Blower SM, Hartel D, Dowlatabadi H, Anderson RM, May RM (1991). Drugs, sex and HIV: a mathematical model for New York City. Philos Trans R Soc Lond B Biol Sci.

[B12] Hueck CJ (1998). Type III protein secretion systems in bacterial pathogens of animals and plants. Microbiol Mol Biol Rev.

[B13] Hsia RC, Pannekoek Y, Ingerowski E, Bavoil PM (1997). Type III secretion genes identify a putative virulence locus of Chlamydia. Molecular microbiology.

[B14] Fields KA, Hackstadt T (2000). Evidence for the secretion of Chlamydia trachomatis CopN by a type III secretion mechanism. Molecular microbiology.

[B15] Fields KA, Mead DJ, Dooley CA, Hackstadt T (2003). Chlamydia trachomatis type III secretion: evidence for a functional apparatus during early-cycle development. Molecular microbiology.

[B16] Matsumoto A (1981). Isolation and electron microscopic observations of intracytoplasmic inclusions containing Chlamydia psittaci. Journal of bacteriology.

[B17] Hackstadt T, Fischer ER, Scidmore MA, Rockey DD, Heinzen RA (1997). Origins and functions of the chlamydial inclusion. Trends Microbiol.

[B18] Winstanley C, Hart CA (2001). Type III secretion systems and pathogenicity islands. J Med Microbiol.

[B19] Bavoil PM, Hsia R, Ojcius DM (2000). Closing in on Chlamydia and its intracellular bag of tricks. Microbiology.

[B20] Zhang YX, Stewart S, Joseph T, Taylor HR, Caldwell HD (1987). Protective monoclonal antibodies recognize epitopes located on the major outer membrane protein of Chlamydia trachomatis. J Immunol.

[B21] Hessel T, Dhital SP, Plank R, Dean D (2001). Immune response to chlamydial 60-kilodalton heat shock protein in tears from Nepali trachoma patients. Infection and immunity.

[B22] LaVerda D, Kalayoglu MV, Byrne GI (1999). Chlamydial heat shock proteins and disease pathology: new paradigms for old problems?. Infect Dis Obstet Gynecol.

[B23] Kinnunen A, Paavonen J, Surcel HM (2001). Heat shock protein 60 specific T-cell response in chlamydial infections. Scand J Immunol.

[B24] Suchland RJ, Rockey DD, Bannantine JP, Stamm WE (2000). Isolates of Chlamydia trachomatis that occupy nonfusogenic inclusions lack IncA, a protein localized to the inclusion membrane. Infect Immun.

[B25] Crepin VF, Shaw R, Abe CM, Knutton S, Frankel G (2005). Polarity of enteropathogenic Escherichia coli EspA filament assembly and protein secretion. Journal of bacteriology.

[B26] Marlovits TC, Kubori T, Lara-Tejero M, Thomas D, Unger VM, Galan JE (2006). Assembly of the inner rod determines needle length in the type III secretion injectisome. Nature.

[B27] Mota LJ, Journet L, Sorg I, Agrain C, Cornelis GR (2005). Bacterial injectisomes: needle length does matter. Science.

[B28] Tamano K, Katayama E, Toyotome T, Sasakawa C (2002). Shigella Spa32 is an essential secretory protein for functional type III secretion machinery and uniformity of its needle length. Journal of bacteriology.

[B29] Minamino T, Pugsley AP (2005). Measure for measure in the control of type III secretion hook and needle length. Molecular microbiology.

[B30] Rockey DD, Viratyosin W, Bannantine JP, Suchland RJ, Stamm WE (2002). Diversity within inc genes of clinical Chlamydia trachomatis variant isolates that occupy non-fusogenic inclusions. Microbiology.

[B31] Pannekoek Y, Spaargaren J, Langerak AA, Merks J, Morre SA, van der Ende A (2005). Interrelationship between polymorphisms of incA, fusogenic properties of Chlamydia trachomatis strains, and clinical manifestations in patients in The Netherlands. Journal of clinical microbiology.

[B32] Matsumoto A (1973). Fine structures of cell envelopes of Chlamydia organisms as revealed by freeze-etching and negative staining techniques. Journal of bacteriology.

[B33] Matsumoto A, Fujiwara E, Higashi N (1976). Observations of the surface projections of infectious small cell of Chlamydia psittaci in thin sections. J Electron Microsc (Tokyo).

[B34] Gregory WW, Gardner M, Byrne GI, Moulder JW (1979). Arrays of hemispheric surface projections on Chlamydia psittaci and Chlamydia trachomatis observed by scanning electron microscopy. Journal of bacteriology.

[B35] Miyashita N, Kanamoto Y, Matsumoto A (1993). The morphology of Chlamydia pneumoniae. J Med Microbiol.

[B36] Peterson EM, de la Maza LM (1988). Chlamydia parasitism: ultrastructural characterization of the interaction between the chlamydial cell envelope and the host cell. Journal of bacteriology.

[B37] Soloff BL, Rank RG, Barron AL (1982). Ultrastructural studies of chlamydial infection in guinea-pig urogenital tract. Journal of comparative pathology.

[B38] Ward ME (1983). Chlamydial classification, development and structure. Br Med Bull.

