# Supplementary material for: Spatial constraints within the chlamydial host cell inclusion predict interrupted development and persistence
Source: BMC Microbiol. 2008 Jan 9;8:5. doi: 10.1186/1471-2180-8-5 (PMC2254404; doi:10.1186/1471-2180-8-5)
Supplement: Additional file 1 — Details of modeling methodology and algorithms. Provides detailed information to describe the mathematical modelling methodology (including deterministic equations, geometry, and algorithms for stochastic simulations). [file 1471-2180-8-5-S1.doc]

**Details of modeling methodology and algorithms**

Table of Contents

[1. Overview of methods (model structure) 2](#__RefHeading___Toc181681191)

[1.1 Deterministic Model 2](#__RefHeading___Toc181681192)

[1.2 Stochastic Model 4](#__RefHeading___Toc181681192)

2. Geometry of RB-inclusion interactions 7

[3. Stochastic model equations 11](#__RefHeading___Toc181681193)

[4. Algorithm/pseudo-code for stochastic simulations 15](#__RefHeading___Toc181681194)

References 20

# 1. Overview of methods (model structure)

**1.1 Deterministic Model**

We track the number of attached RBs,$R(t)$, detached RBs,$I(t)$, and EBs, $E(t)$. The populations of each of these types of particles changes with time as described by the following three differential equations (one for each particle type):

$\frac{dR}{dt}=\frac{\ln2}{t_{d}}R\left(1-\frac{R}{R_{\max}}\right)-\delta(t)R$ (1)

$\frac{dI}{dt}=\delta(t)R-\mu I$ (2)

$\frac{dE}{dt}=\mu I$. (3)

Here, the left hand side of the equations is the time derivative representing the rate of change of these populations and the right hand sides of the equations describe what influences change in the population numbers. The rate of growth of RBs is influenced by the doubling time, $t_{d}$, and saturates to the maximum number that can fit within the inclusion due to cell spatial constraints, $R_{\max}$, and the rate that detached RBs differentiate into EBs is represented by *μ*. We use geometry to develop an expression for $R_{\max}$ based on the physical properties of the RB and the inclusion, and this is given by

$R_{\max}=\frac{\pi\left(\rho_{\max}-r\right)}{r\theta}$, (4)

where

$\theta=\arcsin\left(\frac{r}{\rho_{\max}-r}\right)$. (5)

Here, $r$ is the average radius of the RBs, is the maximum radius to which each inclusion may grow, Cell_Vol_ represents the volume of the host cell, *ε* the proportion of the cell volume taken up by the cell nucleus and mitochondria, and $N$ is the number of inclusions within the infected cell. See Section 2 for further description including all geometric expressions and see Table 1 in the main text for definitions and values of all model parameters. Detachment of RBs from the CIM occurs at the rate$\delta(t)$, expressed by

$\delta(t)=\left\{\begin{matrix} k\left(\frac{P_{\lim}-P(t)}{P(t)}\right), & P(t)\leq P_{\lim} \\ 0 & P(t)>P_{\lim} \end{matrix}\right.$, (6)

where 1/*k* is the average time for RB detachment from the inclusion membrane when the number of T3S needles per RB is half the threshold level, $P_{\lim}$ is the threshold number of T3S needles per RB for detachment from the CIM to occur and $P(t)=p_{s}\alpha$ is the time-dependent number of T3S needles per RB, given the density of projections on the RB surface ($p_{s}$) and the surface area in contact with the CIM ($\alpha$). The surface area in contact is calculated geometrically, and dynamically, based on the radius of the inclusion ($\rho$), the RB radius ($r$), and the length of each needle ($l_{p}$) that is protruding from the RB to the inclusion membrane to establish contact, and is given by

$\alpha=\frac{\pi\rho l_{p}\left(2r+l_{p}\right)}{\rho-r}$. (7)

The density of T3S needles,$p_{s}$, is the number of surface projections per square micron on the RB surface and is given by$p_{s}=1/L^{2}$ ($L$ is the average center-to-center spacing between projections on the RB surface (measured by Matsumoto [1])). We note that the number of T3S needles per RB determined by our geometric algorithm during our model simulations was found to be completely consistent with the range of surface projections measured by Matsumoto experimentally [2] (results not shown). The radius of the inclusion,$\rho$, is taken to depend on the total number of chlamydial particles within the inclusion, $S(t)=R(t)+I(t)+E(t)$, and expressed implicitly as

$rS(t)\arcsin\left(\frac{r}{\rho-r}\right)=\pi\left(\rho-r\right)$, (8)

consistent with equation 4, but if *ρ*>>*r* then

$\rho\approx r\left(1+\sqrt{\frac{S(t)}{\pi}}\right)$. (9)

The rate of RB detachment from the CIM is zero until the threshold is reached and then increases as the number of T3S needles decreases. Detached RBs differentiate into EBs after an average of $1/\mu$hours.

We calculated a threshold for defining whether persistence growth or normal development would ensue. Persistence will occur if the average number of T3S needles per RB,$P(t)$, never decreases below the threshold$P_{\lim}$. Thus, the threshold is$P(t)=P_{\lim}$. Here, the steady state RB level is $R_{\max}$ and the threshold expression is solved implicitly using equations (8); but if *ρ*>>*r* the threshold is given by

$P_{\lim}=p_{s}\pi l_{p}\left(2r+l_{p}\right)\left[1+\sqrt{\frac{r}{\Omega}\arcsin\left(\frac{r}{\Omega}\right)}\right]$ (10)

where . Persistence will occur if$P^{*}>P_{\lim}$ and normal development will occur if $P^{*}<P_{\lim}$. We modeled that cell lysis could also occur if the inclusion volume is filled completely with chlamydial bodies such that it expands more than the total cell volume or if the total T3S level (as indicated by the total number of needles in contact with the CIM across all RBs) drops below a threshold (ranging 600-1000).

**1.2 Stochastic Model**

Deterministic models are appropriate when the population numbers being tracked are relatively large. However, stochastic features allow more accurate tracking of small populations. This is important in the persistent mode of growth. We convert our deterministic differential equation model into an equivalent stochastic model to more appropriately accommodate the uncertainty inherent in the development, especially for low numbers of particles in persistent cases. We also incorporate considerably greater detail. Our model tracks the number of attached RBs, detached RBs, and EBs in all inclusions of an infected cell. There are three basic stochastic events that can occur for these chlamydial bodies during development within an inclusion: (i) RBs can replicate; (ii) RBs can detach from the inclusion surface; (iii) detached RBs can differentiate to EBs. Since each event results in one of two possible outcomes (that is, an RB replicates or does not replicate; an RB detaches or it remains attached to the inclusion; a detached RB differentiates into an EB or remains an RB), we have a Bernoulli trial for each event. The binomial distribution arises from repeating these independent Bernoulli trials. Thus, at every (small) time step of our stochastic simulations we sample from the binomial distribution with appropriate parameters equivalent to the deterministic model. Another stochastic event that can occur in the system is RBs with multiple genomes (such as newly replicated RBs or maxi-RBs) can divide into distinct bodies. Replicating RBs will divide in the absence of stress and in the presence of stress they may form enlarged aberrant forms, possibly with multiple chromosomes, upon continued replication. We specifically track RBs with one chromosome, RBs with two chromosomes, RBs with three chromosomes, etc and explicitly model the stochastic event of these multi-nucleated RBs dividing, in order to accurately capture the behavior of persistent infection when multi-nucleated RBs do not divide. We also investigated the random cases of inclusion division, fusion of multiple inclusions, and host cells dividing; we carried out a detailed investigation of these cases but found that they did not significantly alter outcomes (results not shown). We carried out 10,000 simulations of the chlamydial developmental time course; for each simulation a different set of parameters were chosen from the appropriate parameter space (specified in Table 1) based on the Latin Hypercube Sample. This procedure was repeated systematically by changing the number of inclusions from one to twenty. Refer to Section 4 for full mathematical algorithms and details of our stochastic model.

2. Geometry of RB-inclusion interactions

We now provide detailed descriptions of all geometric features incorporated in our mathematical model. Definitions of all geometric parameters are listed in Table S1.

**Maximum number of RBs within inclusion, *R*_max_**

To calculate$R_{\max}$, the maximum number of RBs of radius $r$ that can fit inside an inclusion of radius $\rho_{\max}$, we first estimate the two-dimensional case of the number of circles of radius $r$that can fit in a circle of radius$\rho_{\max}$. Each RB connected to the inclusion forms a sector defined by angle $\theta$ as shown in Figure S1. Using triangle ABC in Figure S1, the angle *θ* is calculated to be

$\theta=2\arcsin\left(\frac{r}{\rho_{\max}-r}\right)$. (S1.1)

The volume of the wedge (Fig. S1a) can then be calculated, using polar coordinates:

$\begin{matrix} V_{\text{wedge}}=\int_{\frac{\pi-\theta}{2}}^{\frac{\pi+\theta}{2}} \int_{0}^{\theta} \int_{\rho_{\max}-2r}^{\rho_{\max}} r^{2}\sin\varphi\text{ d}r\text{d}\theta\text{d}\varphi\\ =\frac{2r\theta}{3(\rho_{\max}-r)}\left(\rho_{\max}^{3}-(\rho_{\max}-2r)^{3}\right). \end{matrix}$ (S1.2)

It follows that the volume of the spherical ‘shell’ housing the RBs is

$V_{\text{shell}}=\frac{4\pi}{3}\left(\rho_{\max}^{3}-(\rho_{\max}-2r)^{3}\right)$. (S1.3)

Then the maximum number of RBs of radius $r$to fit in an inclusion with radius $\rho_{\max}$ is the quotient of $V_{\text{shell}}$ and $V_{\text{wedge}}$, namely,

$R_{\max}=\frac{2\pi\left(\rho_{\max}-r\right)}{r\theta}$. (S1.4)

| **a)**  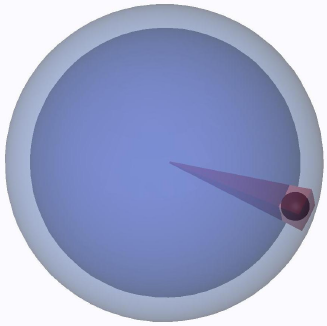 | **b)**  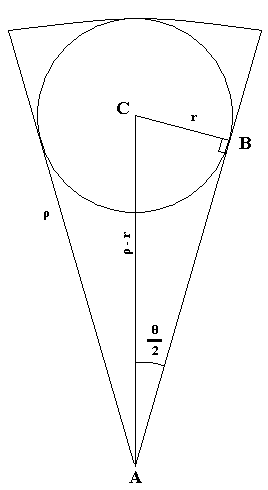 |
| --- | --- |

Figure S1: The number of spheres to fit in the inner boundary layer of a larger sphere (a) is calculated by reducing the problem to (b) the two-dimensional sector defined by the largest cross-sectional circles. The inclusion has radius *ρ* and it contains a RB of radius *r*.

**
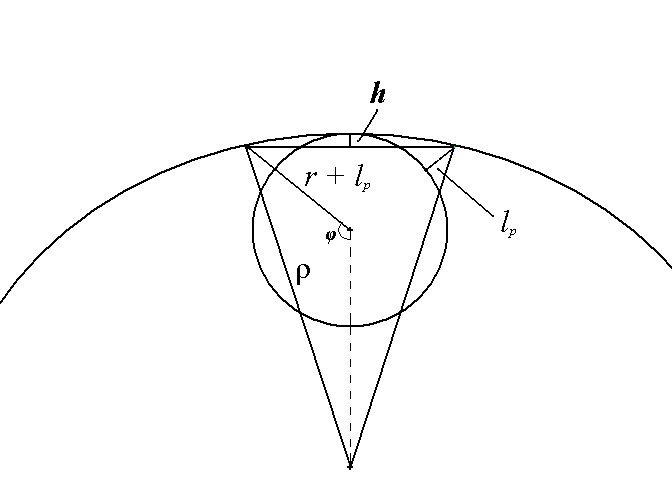
**

**Figure S2: Geometry connecting the RB, inclusion, and RB projection.**

**Surface area in contact between RB and inclusion membrane, *α***

The surface area of the inclusion membrane in contact with each RB is calculated geometrically based on the radius of the inclusion ($\rho$), the RB radius ($r$), and the length of each projection ($l_{p}$) that is protruding from the RB to the inclusion membrane to establish contact. To determine the surface area of the ‘cap’ we use the formula: $\alpha=2\pi\rho h$ where *h* is the height of the ‘cap’, which we must find using geometry (see Fig. S2). To calculate *h*, the angle $\phi$ must be determined (in Fig. S2). Using elementary trigonometry we have,

$\phi=\arccos\left(\frac{\left(\rho-r\right)^{2}+\left(r+l_{p}\right)^{2}-\rho^{2}}{2\left(\rho-r\right)\left(r+l_{p}\right)}\right)$, (S1.5)

leading to

$h=r+\frac{\left(\rho-r\right)^{2}+\left(r+l_{p}\right)^{2}-\rho^{2}}{2\left(\rho-r\right)}$ (S1.6)

and thus the surface area required is

$\alpha=\frac{\pi\rho l_{p}\left(2r+l_{p}\right)}{\rho-r}$. (S1.7)

**Radius of inclusion, *ρ***

The radius of the inclusion depends on the number of chlamydial bodies,$S(t)$, within the host inclusion. The relationship between chlamydial bodies and the inclusion radius has already been determined (equations (S1.1) and (S1.4)), namely,

$S(t)=\frac{\pi\left(\rho-r\right)}{r\arcsin\left(\frac{r}{\rho-r}\right)}$. (S1.8)

Since there is no closed-form solution of equation (S1.8) for$\rho$, we must solve it numerically and dynamically. However, for$\rho>>r$, $\arcsin\left(\frac{r}{\rho-r}\right)\approx\frac{r}{\rho-r}$ and the radius of the inclusion can be very well approximated by

$\rho\approx r\left(1+\sqrt{\frac{S(t)}{\pi}}\right)$. (S1.9)

Table S1: Description of parameters used in the geometric components of the model

| **Parameter Name** | **Description of Parameter** |
| --- | --- |
| $r$ | Radius of RB |
| $\rho$ | Radius of Inclusion |
| $\rho_{\max}$ | Maximum allowable radius for an inclusion |
| $\theta$ | Angle of sector containing an RB in a 2D plane |
| $l_{p}$ | Length of T3S needles |
| $\alpha$ | Surface area in contact between RB and inclusion membrane |
| $S(t)$ | Total number of chlamydial bodies (*R(t)+I(t)+E(t)*) |

# 3. Stochastic model equations

We model the number of attached RBs$\left(R_{cij}(t)\right)$, detached RBs$\left(I_{cij}(t)\right)$, and EBs $\left(E_{cij}(t)\right)$ in cell ‘*c*’ and inclusion ‘$i$’ at time $t$ post infection (index *j* refers to the number of chromosomes per chlamydial body, to account for chlamydial bodies that are replicating their DNA but are not dividing (especially in the special case of stress-induced persistent maxi-RBs)). To determine the size of inclusion ‘$i$’, at time$t$, we use the cumulative number of RBs (chromosome equivalents) that have been in the inclusion prior to time $t$ (which is equivalent to the total number of chlamydial chromosomes at time$t$), namely,

$S_{ci}(t)=\sum_{j} j\cdot\left[R_{cij}(t)+I_{cij}(t)+E_{cij}(t)\right]$. (S2.1)

The radius of the inclusion,$\rho_{ci}(t)$, and the maximum number of RBs that may exist simultaneously in inclusion $i$,$R_{ci}^{\max}(t)$, are determined as described in the deterministic model section.

We make allowances for multiple inclusions per host cell$\left(N_{c}\right)$; any inclusion may potentially divide and so the number of inclusions per host cell can change dynamically. The maximum volume available for EBs and detached RBs in inclusion ‘$i$’ (of cell ‘*c*’) is

$V_{ci}^{\max}(t)=\frac{4}{3}\pi\left(\rho_{ci}(t)-2r\right)^{3}$, (S2.2)

where RBs occupy a boundary layer within the inside boundary of each inclusion, and the volume of space occupied and excluded by detached RBs and EBs in inclusion ‘$i$’,$V_{ci}(t)$, is given by

$V_{ci}(t)=V_{I}I_{ci}(t)+V_{E}E_{ci}(t)$, (S2.3)

where $V_{I}$ and $V_{E}$ are the volumes of space made effectively unavailable per detached RB and EB respectively, considering steric hindrance factors. The maximum volume of cell space available for inclusion $i$ to grow is the available space in cell ‘*c*’ at time $t$, denoted by$A_{ci}(t)$. This available space for inclusion *i* to grow changes with time and is expressed by

$A_{ci}(t)=\text{Cell}_{\text{Vol}}(1-\varepsilon)-\frac{4}{3}\pi\sum_{k=1,k\neq i}^{N_{c}} \rho_{ck}(t)^{3}$, (S2.4)

where $\text{Cell}_{\text{Vol}}$ is the volume of the host cell and $\varepsilon$ is the proportion of space within the cell that is unavailable for inclusion growth due to occupation by organelles such as the cell nucleus and mitochondria. Accordingly, based on the available cell space remaining, the maximum radius allowable for inclusion ‘$i$’ is

. (S2.5)

The area of each RB surface that is in contact with the inclusion membrane, $\alpha_{cij}$, is given by

$\alpha_{cij}=\frac{\pi\rho_{cij}l_{p}\left(2r_{j}+l_{p}\right)}{\rho_{cij}-r_{j}}$, (S2.6)

where  is the radius of a chlamydial body with $j$chromosomes and $r$is the radius of a single-chromosome RB. Then, the number of RB projections per RB (with *j* chromosomes) in contact with inclusion membrane *i*, $P_{cij}$, is given by

$P_{cij}=p_{s}\cdot\alpha_{cij}$. (S2.7)

Changes in the attached RB, detached RB, and EB populations are governed by:

$R_{cij}\left(t+\Delta t\right)=R_{cij}\left(t\right)+\left(\sum_{k=\left\lceil\frac{j}{2}\right\rceil}^{j-1} \xi_{1}^{i,k\to j}\right)-\left(\sum_{k=j+1}^{2j} \xi_{1}^{i,j\to k}\right)-\xi_{2}^{ij}$ (S2.8)

$I_{cij}\left(t+\Delta t\right)=I_{cij}\left(t\right)+\xi_{2}^{ij}-\xi_{3}^{ij}$ (S2.9)

$E_{cij}\left(t+\Delta t\right)=E_{cij}\left(t\right)+\xi_{3}^{ij}$, (S2.10)

where, in time step $\left(t,t+\Delta t\right]$, $\xi_{1}^{i,k\to j}$ refers to the number of RBs with *k* chromosomes that become RBs with *j* chromosomes, $\xi_{2}^{ij}$ refers to the number of RBs that detach from the inclusion membrane, and $\xi_{3}^{ij}$refers to the number of detached RBs that complete differentiation into EBs.

To calculate$\xi_{1}^{i,k\to j}$, the change in RBs due to replication and division, we create a set *S* of size equal to the number of RBs and for each body we sample the probability of replication and division events from a binomial distribution. The probability that an individual RB will replicate its DNA in a time interval of $\Delta t$ is given by:

$\Delta t\frac{\ln(2)}{t_{d}}\left(1-\frac{\sum_{m} mR_{cim}}{R_{ci}^{\max}}\right)$. (S2.11)

where $\ln(2)/t_{d}$ is the unconstrained rate of replication of RBs and $t_{d}$ is the average doubling time. The production of new RBs is limited by the available space potentially remaining on the inclusion membrane, $R_{ci}^{\max}$. The set *S* is defined as

$S=\left\{x_{k},k=1...R_{cij}\left(t\right)|x_{k}\sim B\left(j,\Delta t\frac{\ln\left(2\right)}{t_{d}}\left(1-\frac{\sum_{m} mR_{cim}}{R_{ci}^{\max}}\right)\psi_{o}\right)\right\}$, (S2.12)

where *j* is the number of chromosomes. We include the factor *ψ_0_*, to take into account the spatial limitations of the cell, acting as a switch; $\psi_{0}=0$ when the volume occupied is the total volume available and $\psi_{0}=1$ otherwise. Then, $\xi_{1}^{i,k\to j}$ is defined as

$\xi_{1}^{ij\to k}=\#\left\{x:S|x=k-j\right\},k=j+1...2j$. (S2.13)

To calculate$\xi_{2}^{ij}$, the number of RBs that detach from the inclusion membrane, we convert the deterministic rate of RB detachment,

$\delta(t)=\left\{\begin{matrix} k\left(\frac{P_{\lim}-P(t)}{P(t)}\right), & P(t)\leq P_{\lim} \\ 0 & P(t)>P_{\lim} \end{matrix}\right.$,

into a stochastic equivalent. The probability of detachment per RB per time step $\Delta t$ is $\Delta t\delta_{cij}\psi_{1}$, where $\psi_{1}$ acts as a switch (from 1 to 0) if the inclusion lumen fills to capacity. Then $\xi_{2}^{ij}$ is defined as

$\xi_{2}^{ij}\sim B\left(R_{cij}-\sum_{k=j+1}^{2j} \xi_{1}^{j\to k},\Delta t\delta_{cij}\psi_{1}\right)$. (S2.14)

Here, only RBs that have not replicated or divided in the given time interval can detach from the inclusion membrane; that is, only a single event can occur per chlamydial body over any short time interval $\Delta t$.

Similarly, the probability that a detached RB will differentiate into an EB is$\Delta t\mu\Lambda_{1j}$, where *μ^-1^* is the average time for RB-to-EB differentiation, and $\Lambda_{1j}$ is the Kronecker delta function to ensure that only mono-nucleated RBs can differentiate to EBs. We then define $\xi_{3}^{ij}$as

$\xi_{3}^{ij}\sim B\left(I_{cij},\Delta t\mu\Lambda_{1j}\right)$, (S2.15)

where *I_cij_* is the number of detached RBs.

# 4. Algorithm/pseudo-code for stochastic simulations

Here, we provide pseudo-code for the implementation of our stochastic model. All parameters are defined in the previous section and in Table S2.

**For** $t\leftarrow\Delta t\ldots T\text{ (by }\Delta t\text{)}$

**For** $c\leftarrow1\ldots N$

For $i\leftarrow1\ldots N_{c}$

For $j\leftarrow1\ldots N_{g}$ ($N_{g}$ is the maximum number of chromosomes per chlamydial body)

**Modeling the basic stochastic events of chlamydial development within an inclusion**

$\psi_{0}=H\left(V_{ci}^{\max}-V_{ci}\right)$; where *H* is the Heaviside step function.

$$\psi_{1}=H\left(V_{ci}^{\max}-V_{ci}\right)H\left(\rho_{ci}(t)-3r\right)$$

$$\delta_{cij}=k\left(\frac{P_{\lim}-P_{cij}}{P_{cij}}\right)H\left(P_{\lim}-P_{cij}\right)$$

$$S=\left\{\left.x_{k},k=1\ldots R_{cij}(t)\right|x_{k}\sim B\left(j,\Delta t\frac{\ln(2)}{t_{d}}\left(1-\frac{\sum_{m} m\cdot R_{cim}}{R_{ci}^{\max}}\right)\cdot\psi_{0}\right)\right\}$$

$\xi_{1}^{i,j\to k}=\#\left\{\left.x:S\right|x=k-j\right\}$, $k\leftarrow j+1\ldots2j$

$$\xi_{2}^{ij}\sim B\left(R_{cij}-\sum_{k=j+1}^{2j} \xi_{1}^{j\to k},\Delta t\delta_{cij}\psi_{1}\right)$$

$\xi_{3}^{i,j}\sim B\left(I_{cij},\Delta t\mu\Lambda_{1,j}\right)$
**Change the chlamydial numbers due to basic stochastic events**

$$R_{cij}(t+\Delta t)=R_{cij}(t)+\left(\sum_{k=\left\lceil\frac{j}{2}\right\rceil}^{j-1} \xi_{1}^{i,k\to j}\right)-\left(\sum_{k=j+1}^{2j} \xi_{1}^{i,j\to k}\right)-\xi_{2}^{i,j}$$

$$I_{cij}(t+\Delta t)=I_{cij}(t)+\xi_{2}^{i,j}-\xi_{3}^{i,j}$$

$$E_{cij}(t+\Delta t)=E_{cij}(t)+\xi_{3}^{i,j}$$

End *j*-for

End *i*-for

**Modeling the division of chlamydial chromosomes**

Attached RBs

For $i\leftarrow1\ldots N_{c}$

$$s=\bigcup_{j} \left\{j,k=1\ldots R_{cij}\right\}$$

For $j\leftarrow2\ldots N_{g}$

For $k\leftarrow1\ldots R_{cij}(t+\Delta t)$

$$s=s-\left\{j\right\}$$

$$D'\sim B\left(j-1,\lambda_{R}\Delta t\right)$$

$$D_{I}\sim B\left(1,\varphi_{ciD'}H\left(\rho_{ci}(t)-3r\right)\right)$$

$$s=s\cup\left\{j-D'\right\}$$

End *k*-for

End *j*-for

For $j\leftarrow1\ldots N_{g}$

$$R_{cij}(t+\Delta t)=\#\left\{\left.x:s\right|x=j\right\}$$

$$I_{cij}=I_{cij}+\#\left\{\left.x:s_{i}^{R\to I}\right|x=j\right\}$$

End *j*-for

End *i*-for

Detached RBs

For $i\leftarrow1\ldots N_{c}$

$$s=\bigcup_{j} \left\{j,k=1\ldots I_{cij}\right\}$$

For $j\leftarrow2\ldots N_{g}$

For $k\leftarrow1\ldots I_{cij}(t+\Delta t)$

$$s=s-\left\{j\right\}$$

$$D'\sim B\left(j-1,\lambda_{I}\right)$$

$$s=s\cup\left\{j-D',D'\right\}$$

End *k*-for

End *j*-for

For $j\leftarrow1\ldots N_{g}$

$$I_{cij}(t+\Delta t)=\#\left\{\left.x:s\right|x=j\right\}$$

End *j*-for

End *i*-for

**Modeling the division of inclusions**

For $i\leftarrow1\ldots N_{c}$

Inclusion ‘*i*’ divides with probability$\Delta t\frac{1}{T_{I}^{d}}\left(1-\frac{\sum_{m} m\cdot R_{cim}}{R_{ci}^{\max}}\right)$, where $T_{I}^{d}$is the average time for inclusions to divide during maximal chlamydial expansion.

If inclusion ‘*i*’ divides then

$$s_{R}=\bigcup_{j} \left\{j,k=1\ldots R_{cij}\right\}$$

For $k\leftarrow1\ldots B\left(\sum_{j} R_{cij},0.5\right)$

$$b\in s_{R}$$

$$s_{R}=s_{R}-\left\{b\right\}$$

End *k*-for

$$s_{I}=\bigcup_{j} \left\{j,k=1\ldots I_{cij}\right\}$$

For $k\leftarrow1\ldots B\left(\sum_{j} I_{cij},0.5\right)$

$$b\in s_{I}$$

$$s_{I}=s_{I}-\left\{b\right\}$$

End *k*-for

$$s_{E}=\bigcup_{j} \left\{j,k=1\ldots E_{cij}\right\}$$

For $k\leftarrow1\ldots B\left(\sum_{j} E_{cij},0.5\right)$

$$b\in s_{E}$$

$$s_{E}=s_{E}-\left\{b\right\}$$

End *k*-for

$$N_{c}=N_{c}+1$$

$$t_{ci}^{0}=t$$

$$t_{cN_{c}}^{0}=t$$

For $j\leftarrow1\ldots N_{g}$

$$R_{cij}=\#\left\{\left.x:s_{R}\right|x=j\right\}$$

$$I_{cij}=\#\left\{\left.x:s_{I}\right|x=j\right\}$$

$$E_{cij}=\#\left\{\left.x:s_{E}\right|x=j\right\}$$

End *j*-for

End-if

End *i*-for

**Modeling the fusion of inclusions**

A fusion event (between two of the $N_{c}$ inclusions in cell ‘*c*’) will occur in the time interval$t\to t+\Delta t$ with probability $\Delta t\frac{1}{T_{I}^{f}}\prod_{k=1}^{N_{c}} \left(1-\frac{\sum_{m} m\cdot R_{ckm}}{R_{ck}^{\max}}\right)$, where the average time between fusing events during maximal chlamydial expansion is $T_{I}^{f}$.

If fusion occurs then

Randomly choose which two (of the$N_{c}$) inclusions fuse together: inclusions $i_{1}$ and $i_{2}$

For $j\leftarrow1\ldots N_{g}$

$$R_{ci_{1}j}=R_{ci_{1}j}+R_{ci_{2}j}$$

$$I_{ci_{1}j}=I_{ci_{1}j}+I_{ci_{2}j}$$

$$E_{ci_{1}j}=E_{ci_{1}j}+E_{ci_{2}j}$$

End *j*-for

$$t_{ci_{1}}^{0}=t$$

For $i\leftarrow i_{2}+1\ldots N_{c}$

For $j\leftarrow1\ldots N_{g}$

$$R_{c,i-1,j}=R_{cij}$$

$$I_{c,i-1,j}=I_{cij}$$

$$E_{c,i-1,j}=E_{cij}$$

End *j*-for

End *i*-for

$$N_{c}=N_{c}-1$$

End-if

End *c*-for

End *t*-for

Table S2: Description of parameters used in the stochastic model

| **Parameters** | **Description of Parameter** |
| --- | --- |
| $R_{cij}(t)$ | Number of attached RBs at time *t*, in cell *c*, in inclusion *i*, of *j* chromosomes |
| $I_{cij}(t)$ | Number of detached RBs at time *t*, in cell *c*, in inclusion *i*, of *j* chromosomes |
| $E_{cij}(t)$ | Number of EBs at time *t*, in cell *c*, in inclusion *i*, of *j* chromosomes |
| $S_{ci}(t)$ | Total number of chlamydial chromosomes |
| $\rho_{ci}(t)$ | Radius of inclusion *i*, in cell *c*, at time *t*. |
| $R_{ci}^{\max}(t)$ | Maximum number of RBs for inclusion *I*, in cell *c*, at time *t*. |
| $N_{c}$ | Number of inclusions in cell *c* |
| $V_{ci}^{\max}(t)$ | Maximum volume of inclusion *i*, in cell *c*, at time *t* |
| $V_{ci}(t)$ | Volume of inclusion *i*, in cell *c*, at time *t*, occupied by detached RBs and EBs |
| $V_{I}$ | Volume occupied be detached RBs |
| $V_{E}$ | Volume occupied be detached EBs |
| $A_{ci}(t)$ | Maximum volume available for inclusion *I*, in cell *c*, to grow into |
| $\text{Cell}_{\text{Vol}}$ | Volume of the host cell |
| $\varepsilon$ | Proportion of host cell unavailable for inclusion growth |
| $\rho_{ci}^{\max}(t)$ | Maximum radius for inclusion *i*, in cell *c* at time *t* |
| $\alpha_{cij}$ | Surface area of contact between an RB of cell *c*, in inclusion *i*, with *j* chromosomes |
| $l_{p}$ | Length of the T3S projections |
| $r_{j}$ | Radius of RB with *j* chromosomes |
| $r$ | Radius of single chromosome RB |
| $P_{cij}$ | Number of T3S projections per RB |
| $p_{s}$ | Density of RB projections |
| $\xi_{1}^{i,k\to j}$ | Number of RBs with *k* chromosomes that become RBs with *j* chromosomes |
| $\xi_{2}^{ij}$ | Number of RBs that detach from the inclusion membrane |
| $\Delta t$ | Time step of simulations |
| $T$ | End time of tracking developmental cycle |
| $t_{d}$ | Average doubling time of RB growth |
| $\psi_{0},\psi_{1}$ | Heaviside functions used as switches, they are either 1 or 0 |
| $P_{\lim}$ | Threshold number of RB projections required for Rbs to remain attached to inclusion membrane |
| $\delta_{cij}$ | Rate of RB detachment from inclusion membrane |
| $\xi_{3}^{ij}$ | Number of detached RBs that differentiate into EBs |
| $\mu$ | Rate of RB to EB differentiation |
| $\Lambda_{1j}$ | Kronecker delta function, is equal to 1 only if *j* = 1, and is 0 otherwise |

**References**

1. Matsumoto A: **Fine structures of cell envelopes of Chlamydia organisms as revealed by freeze-etching and negative staining techniques**. *Journal of bacteriology* 1973, **116**(3):1355-1363.

2. Matsumoto A: **Electron microscopic observations of surface projections on Chlamydia psittaci reticulate bodies**. *Journal of bacteriology* 1982, **150**(1):358-364.
